# Comparative analysis of 333 proGAV® and proGAV 2.0® adjustable valves in pediatric hydrocephalus treatment: survival and complication rate assessment

**DOI:** 10.1007/s00701-024-06348-9

**Published:** 2024-11-09

**Authors:** Mohammed Issa, Filippo Paggetti, Clara Dannehl, Nieke Ueding, Sandro M. Krieg, Ahmed El Damaty

**Affiliations:** 1 Department of Neurosurgery, Heidelberg University Hospital, Im Neuenheimer Feld 400, 69120 Heidelberg, Germany; 2 Faculty of Medicine, Heidelberg University, Heidelberg, Germany; 3 Department of Neurosurgery, Düsseldorf University Hospital, Düsseldorf, Germany

**Keywords:** ProGAV®, ProGAV 2.0®, Hydrocephalus, Adverse events, Survival and complication rates

## Abstract

**Objective:**

ProGAV and ProGAV2.0 adjustable valves are extensively used for treating hydrocephalus in pediatric patients. This study aims to conduct a comprehensive comparison between these two valves concerning their survival and complication rates.

**Methods:**

This retrospective study included all pediatric patients who underwent ProGAV or ProGAV2.0 valve implantation at our neurosurgical clinic from August 2008 to August 2020. A comparative analysis was performed considering age, gender, follow-up duration, complication and survival rates, adjustments, spontaneous adjustments, and adjustment difficulty rates. All valves were followed up for a maximum of 3 years.

**Results:**

Inclusion involved 333 cases (mean age of 5.4 ± 5.1 years; 54.1% males), comprising 173 cases (52.0%) with ProGAV valve implantation and 160 cases (48.0%) with ProGAV 2.0. Early complications within the first month post-implantation were observed in 51 cases (15.3%). No significant differences were noted in valve implantation indications, age distribution, or survival duration (27.1 vs. 27.8 months, p = 0.629) between the two groups. Predominant indications for implantation were post-hemorrhagic hydrocephalus and aqueduct stenosis for both valve types. Notably, both valves showed non-significantly different explantation rates during the first three years after implantation (34.7% vs. 29.7%, *p* < 0.289). However, there was a significantly higher early post-implantation complication rate (22% vs. 9.4%, ***p* = 0.002**), and a significantly increased association with difficulties in valve adjustments and spontaneous adjustments (***p* = 0.041 and 0.011**, respectively). ProGAV2.0 cases displayed notably enhanced clinical and radiological improvement within the initial 6 months after implantation (***p* = 0.001 and p = 0.038**). Younger children (< 2 years of age) also experienced significantly more valve adjustment difficulties (*p* = **0.049**) and had higher rates of valve explantation (***p* < 0.001**).

**Conclusion:**

The findings of this study highlight the superior performance of the ProGAV2.0 valve in terms of complication rate and maladjustment rate when employed in the treatment of pediatric hydrocephalus. Both valves demonstrated an acceptable survival rate with 65.3% for ProGAV and 71.3% for ProGAV2.0 within three years of implantation.

## Introduction

Hydrocephalus management, particularly in pediatric patients, often relies on the use of programmable shunt valves to regulate cerebrospinal fluid (CSF) flow and prevent complications like overdrainage or underdrainage[4, 8]. Over the years, various valve systems have been developed to optimize treatment outcomes, with the proGAV® and its successor, the proGAV 2.0®, being widely used in clinical practice[9]. While both valves are designed to allow adjustable pressure settings, which are crucial in tailoring CSF drainage to patient-specific needs, there are important differences in their performance[10].

The proGAV valve, introduced in the early 2000s, offered a solution to managing hydrocephalus through its programmable features[15]. However, clinical experience has shown that it can be associated with certain drawbacks, including difficulties in valve adjustments and spontaneous readjustments, which may lead to suboptimal control of CSF drainage and higher complication rates[13, 14]. In response to these issues, the proGAV 2.0 valve was developed with the aim of improving adjustability, reducing complication rates, and enhancing overall valve survival[10].

Several studies have compared the complication rates, survival outcomes, and ease of valve adjustments between these two systems[1–3, 7]. The proGAV 2.0 valve, with its refined design, has demonstrated promising results, with reports of reduced rates of adjustment difficulties and spontaneous changes[5, 10]. However, it remains crucial to examine whether these advancements translate into significantly better clinical outcomes, particularly in terms of complication rates and long-term valve survival.

This study aims to compare the proGAV and proGAV 2.0 valves, focusing on key performance indicators such as valve adjustment difficulties, complication rates, and survival outcomes, to provide further insight into the effectiveness of each valve system in the treatment of pediatric hydrocephalus.

## Methods

### Study design and patient population

This single-center retrospective comparative study was conducted from 2008 to 2023 and included pediatric patients who underwent implantation of either proGAV® or proGAV 2.0® valves at our neurosurgical clinic. Patients were divided into two groups based on the period of valve implantation: the proGAV® group (2008–2014) and the proGAV 2.0® group (2015–2020). The study focused on the outcomes of these two valve types in the treatment of pediatric hydrocephalus. All included patients in both groups were controlled over a fixed period of 36 months since implantation.

### Inclusion and exclusion criteria

We included all patients who received ventriculoperitoneal or ventriculoarterial shunts, between January 2008 and January 2020. Patients who did not have the fixed follow-up period of 36 months were excluded. Patients with tumor-related hydrocephalus were excluded from the analysis due to their survival being more dependent on the oncological condition, as well as the higher likelihood of valve dysfunction resulting from tumor cells in the cerebrospinal fluid (CSF). Informed consent was obtained from all parents or legal guardians, and the study was approved by the institutional ethics committee (S-084/2022).

### Study parameters and outcomes

We collected data on patient demographics, including age and gender, along with the primary outcomes: complication rates, survival rates of the valves, and the need for of valve adjustments, unintended “spontaneous” adjustments, and difficulty with valve adjustments over the fixed follow-up period of 36 months. The objective was to compare valve survival rates using Kaplan–Meier survival analysis and to track the number of shunt revisions. A secondary objective was to identify potential risk factors for valve dysfunction by subgroup analysis based patient age groups (0–2 years, 2–10 years, and 10–18 years). This analysis aimed to support clinical decision-making in valve selection for pediatric hydrocephalus treatment.

Since 2017, antibacterial-impregnated catheters were used in all patients under six months of age and in cases requiring shunt reoperation due to CSF infections. Prior to 2017, the use of such catheters was determined on an individual basis.

### Infection criteria

Infections included CSF, wound, and peritoneal infections, all of which were diagnosed according to established clinical criteria. CSF infections were confirmed through CSF examination, while microbiological analysis was performed on wound and abdominal infection specimens. Pathogen confirmation was required for all infection types.

### Clinical and radiological assessment

Data were collected on the etiology of hydrocephalus, indications for shunt/valve insertion, and preoperative and postoperative clinical and radiological assessments. These included monitoring changes in ventricular size, head circumference, and the frequency of valve pressure adjustments and shunt revisions. Overdrainage was defined based on persistent clinical symptoms (e.g., postural headaches, vomiting, sunken fontanelles, or decreased head circumference) and radiological findings (e.g., slit ventricles or subdural collections) as described by Pedersen et al. A "good clinical outcome" was defined as sustained improvement in preoperative symptoms within the first postoperative year without the need for valve explantation[11]. Additionally, long-term follow-up was used to assess head growth patterns and radiological outcomes, particularly in patients with preoperative slit-ventricle morphology. An improvement in ventricular size postoperatively was considered a positive radiological outcome.

### Statistical analysis

Continuous variables were presented as mean ± standard deviation, and categorical variables as frequencies and percentages. Intergroup comparisons were performed using t-tests for continuous variables and the Mann–Whitney U test or Fisher’s exact test for categorical variables. A p-value of less than 0.05 was considered statistically significant. Statistical analyses and graphical representations were performed using IBM SPSS Statistics 29.

## Results

### Patients’ population and hydrocephalus etiology

A total of 333 cases were included in the study, consisting of 173 patients (52.0%) who received the proGAV® valve and 160 patients (48.0%) who received the proGAV 2.0® valve. The mean age across the cohort was 5.4 ± 5.1 years, with no statistically significant difference between the two groups (proGAV: 5.3 ± 5.2 years; proGAV 2.0: 5.6 ± 5.2 years; *p* = 0.499). However, there was a significant difference in sex distribution, with a higher proportion of males in the proGAV group (60.1%) compared to the proGAV 2.0 group (47.5%, *p* = 0.028).

The median follow-up period for both groups was 36 months. There was no significant difference between the groups in terms of first-time shunt implantation (proGAV: 73%; proGAV 2.0: 81%, *p* = 0.09), indicating that the two groups were comparable in terms of initial surgical management.

In terms of hydrocephalus etiology, post-hemorrhagic hydrocephalus was the most common cause (45.6%) overall, followed by idiopathic aqueduct stenosis (16.2%) and Chiari malformation type II (13.8%). The distribution of hydrocephalus etiologies was similar between the proGAV and proGAV 2.0 groups, with no statistically significant differences.

### Comparative survival analysis

Valve survival rates were comparable between the two groups, with an overall survival rate of 68.2%. The survival rate for the proGAV valve was 65.3%, while the proGAV 2.0 valve showed a slightly higher survival rate of 71.3%, though this difference was not statistically significant (*p* = 0.289). Similarly, the survival duration was not significantly different between the groups (proGAV: 27.1 ± 13.4 months vs. proGAV 2.0: 27.8 ± 11.9 months, *p* = 0.629). However, the time to explantation was significantly longer for the proGAV 2.0 group (8.2 ± 11.9 months) compared to the proGAV group (4.5 ± 8.8 months, *p* = 0.001).

**Fig. 1:**
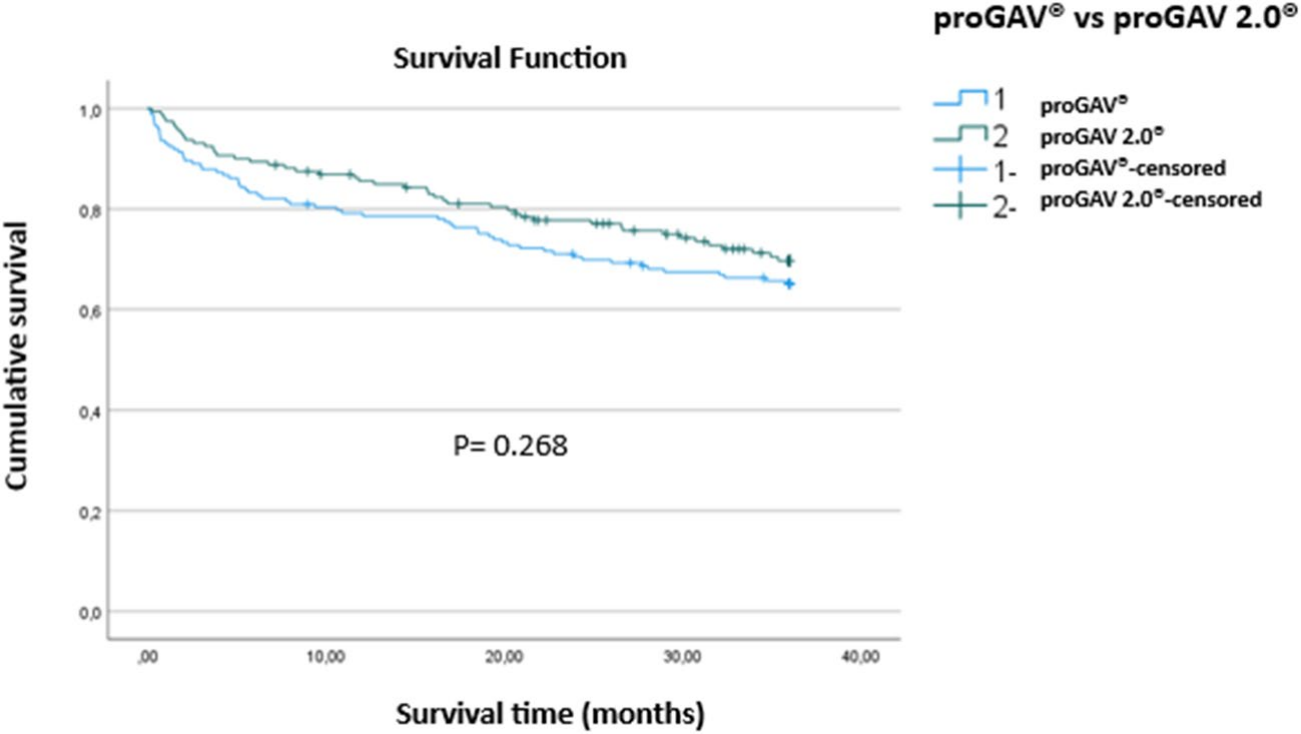
The Kaplan–Meier survival analysis comparing the proGAV and proGAV 2.0 valves revealed difference in survival rates

The Kaplan–Meier (Fig. 1) survival analysis illustrates that the proGAV 2.0 valve demonstrates a slightly better survival rate compared to the proGAV valve over the follow-up period, with a longer mean survival time. Both valves show a gradual decline in survival probability, but the proGAV 2.0 valve maintains a higher probability of survival at key time points, such as 12 and 36 months. However, despite these observations, the difference in survival time between the two valve types is not statistically significant (*p* = 0.268).

### Complication and explantation rate

Postoperative complications (such as ventricular or distal catheter misplacement, wound healing disorder, CSF leakage and shunt disconnection) occurred in 15.3% of the overall cohort, with a significant difference between the groups. Complications were more frequent in the proGAV group, occurring in 22.0% of cases, compared to 9.4% in the proGAV 2.0 group (*p* = 0.002).

Regarding valve explantation, 31.8% of all valves were explanted during the follow-up period, with no significant differences in the overall explantation rates between proGAV (34.7%) and proGAV 2.0 (29.7%, *p* = 0.289). However, when analysing the causes of valve explantation, permanent valve adjustment difficulties were more frequent in the proGAV group (31.2%) compared to the proGAV 2.0 group (18.1%). Other indications for explantation, such as overdrainage, underdrainage, infection, and catheter-related issues, were distributed similarly between the groups and did not reach statistical significance.

### Clinical and radiological outcomes

In terms of clinical improvement, patients in the proGAV 2.0 group showed significantly better outcomes. Within the first 6 months post-implantation, 86.3% of patients in the proGAV 2.0 group demonstrated clinical improvement, compared to 71.7% in the proGAV group (*p* = 0.001). Radiological improvement, defined by changes in ventricular size as intended to be on imaging, was also significantly greater in the proGAV 2.0 group (53.5%) compared to the proGAV group (41.0%, *p* = 0.038).

### Valve data and revisions

Valve adjustment difficulties were more prevalent in the proGAV group, with 31.2% of cases experiencing difficulties, compared to only 18.1% in the proGAV 2.0 group (*p* = 0.011). Additionally, spontaneous valve adjustments occurred more frequently in the proGAV group (33%) compared to the proGAV 2.0 group (23.8%, *p* = 0.041).

Lastly, the number of valve adjustments after implantation was similar between the two groups, with 66.5% of proGAV cases and 63.1% of proGAV 2.0 cases requiring adjustments (*p* = 0.566).

Table 1 highlights significant differences in several clinical outcomes, particularly in terms of postoperative complications, survival rates, and the ease of valve adjustments, suggesting improved performance with the proGAV 2.0 valve.

**Table 1:** Cases characteristics and valve revisions

Groups	Total	proGAV®	proGAV 2.0®	*p*-value**
Number of valves	333 (100)	173 (52)	160 (48)	0.511
Sex Male Female	180 (54.1) 153 (45.9)	104 (60.1) 69 (39.9)	76 (47.5) 84 (52.5)	**0.028**
Mean age (years) ***	5.4 ± 5.1	5.3 ± 5.2	5.6 ± 5.2	0.499
Follow-up (months) ***	72	36	36	1.0
First implantation	256 (77)	126 (73)	130 (81)	0.090
Hydrocephalus etiologies Post-hemorrhagic Idiopathic aqueduct stenosis Chiari Malformation type II Arachnoid cyst Postinfectious Posttraumatic Others	152 (45.6) 54 (16.2) 46 (13.8) 17 (5.1) 15 (4.5) 9 (2.7) 40 (12)	77 (44.5) 26 (15.0) 22 (12.7) 13 (7.5) 9 (5.2) 1 (0.6) 25 (14.4)	75 (46.9) 28 (17.5) 24 (15.0) 4 (2.5) 6 (3.8) 8 (5.0) 15 (9.4)	NS
Overall Survival rate	227 (68.2)	113 (65.3)	114 (71.3)	0.289
Survival time in months	27.4 ± 12.7	27.1 ± 13.4	27.8 ± 11.9	0.629
Time to explant in months	6.3 ± 10.6	4.5 ± 8.8	8.2 ± 11.9	**0.001**
Postoperative complications	51 (15.3)	38 (22.0)	15 (9.4)	**0.002**
Indication for valve explantation Permanent valve adjustment difficulties Overdrainage Underdrainage Infection Catheter-related causes Valve spontaneous adjustments Gravitational (proSA) valve adjustment difficulties Others	106 (31.8) 34 (10.2) 14 (4.2) 18 (5.4) 19 (5.7) 15 (4.5) 3 (0.9) 1 (0.3) 2 (0.6)	60 (34.7) 19 (31.7) 5 (8.3) 11 (18.3) 13 (21.7) 8 (13.3) 2 (3.3) 0 (0.0) 2 (3.3)	46 (29.7) 15 (32.6) 9 (19.6) 7 (15.2) 6 (13.0) 7 (15.2) 1 (2.2) 1 (2.2) 0 (0.0)	0.289 0.055
Clinical improvement within the first 6 month	262 (78.7)	124 (71.7)	138 (86.3)	**0.001**
Radiological improvement within the first 6 months	155 (46.6)	71 (41.0)	84 (53.5)	**0.038**
Adjusted valves after implantation	216 (64.8)	115 (66.5)	101 (63.1)	0.566
Spontaneous valve adjustments	95 (28.5)	57 (33.0)	38 (23.8)	**0.041**
Valve adjustments difficulties	83 (24.9)	54 (31.2)	29 (18.1)	**0.011**

(%) Data in parenthesis are percentages

^**^Bold denotes statistical significance

^***^Data are given as mean ± standard deviation

### Age-Related Comparison of Valve Revisions and Postoperative Outcomes

The study included 333 valves, with a fairly equal distribution among age groups, and no significant differences in valve types (proGAV vs. proGAV 2.0) or gender distribution across the groups. However, a significantly higher rate of first implantations was observed in older children (***p* < 0.001**), while postoperative complications were more frequent in children under 2 years (**23.4%, *p* = 0.012**). Younger children also experienced significantly more valve adjustment difficulties (***p* = 0.049**) and had higher rates of valve explantation due to issues like permanent valve adjustment difficulties and infection (***p* < 0.001**). Clinical and radiological improvements within six months showed no significant age-related differences (*p* = 0.115 and *p* = 0.212, respectively), nor did the rate of spontaneous valve adjustments (*p* = 0.153). Overall, the youngest age group faced greater challenges with postoperative complications and valve management, despite similar clinical and radiological outcomes across age groups. Table 2 compares valve revisions and outcomes across three age groups: under 2 years, 2–10 years, and 10–18 years.

**Table 2:** Age comparison and valve revisions

Groups of Age in years	Total	< 2	2 -10	10—18	*p*-value**
Number of valves	333 (100)	128 (38.4)	132 (39.6)	73 (21.9)	NS
Sex Male Female	180 (54.1) 153 (45.9)	63 (49.2) 65 (50.8)	75 (56.8) 57 (43.2)	42 (57.5) 31 (42.5)	0.385
Valve proGAV proGAV 2.0	173 (52) 160 (48)	72 (56.3) 56 (43.7)	64 (48.5) 68 (41.5)	37 (50.7) 36 (49.3)	0.444
Follow-up (months) ***	108	36	36	36	1.0
First implantation	256 (77)	64 (50)	125 (95)	67 (92)	** < 0.001**
Hydrocephalus etiologies Post-hemorrhagic Idiopathic aqueduct stenosis Chiari Malformation type II Arachnoid cyst Postinfectious Posttraumatic Others	152 (45.6) 54 (16.2) 46 (13.8) 17 (5.1) 15 (4.5) 9 (2.7) 40 (12)	51 (39.8) 23 (18.0) 19 (14.4)) 6 (4.7) 4 (3.1) 5 (3.9) 20 (15.6)	66 (50.0) 17 (12.9) 16 (12.1) 10 (7.6) 8 (6.1) 2 (1.5) 12 (9.1)	35 (43.8) 13 (17.8) 11 (15.1) 1 (1.4) 3 (4.1) 2 (2.7) 8 (11.0)	NS
Postoperative complications	53 (15.9)	30 (23.4)	16 (9.4)	7 (9.6)	**0.012**
Indication for valve explantation Permanent valve adjustment difficulties Overdrainage Underdrainage Infection Catheter-related causes Valve spontaneous adjustments Gravitational (proSA) valve adjustment difficulties Others	106 (31.8) 34 (10.2) 14 (4.2) 18 (5.4) 19 (5.7) 15 (4.5) 3 (0.9) 1 (0.3) 2 (0.6)	61 (47.7) 16 (26.2) 7 (11.5) 14 (23) 14 (23) 6 (9.8) 1 (1.6) 1 (1.6) 2 (3.2)	33(25) 15 (45.5) 5 (15.2) 2 (6.1) 5 (15.2) 5 (15.2) 1 (3.0) 0 (0.0) 0 (0.0)	12 (16.4) 3 (25) 2 (8) 2 (8) 0 (0) 4 (33.3) 1 (8.3) 0	** < 0.001** 0.055
Clinical improvement within the first 6 month	262 (78.7)	93 (72.7)	109 (82.6)	60 (82.2)	0.115
Radiological improvement within the first 6 months	155 (46.6)	66 (51.6)	54 (40.9)	35 (48.0)	0.212
Adjusted valves after implantation	216 (64.8)	87 (68)	83 (62.9)	46 (63)	0.648
Spontaneous valve adjustments	95 (28.5)	34 (26.5)	45 (34.1)	16 (21.9)	0.153
Valve adjustments difficulties	83 (24.9)	41 (32.0)	28 (21.2)	14 (19.2)	**0.049**

(%) Data in parenthesis are percentages

^**^Bold denotes statistical significance

^***^Data are given as mean ± standard deviation

**Fig. 2:**
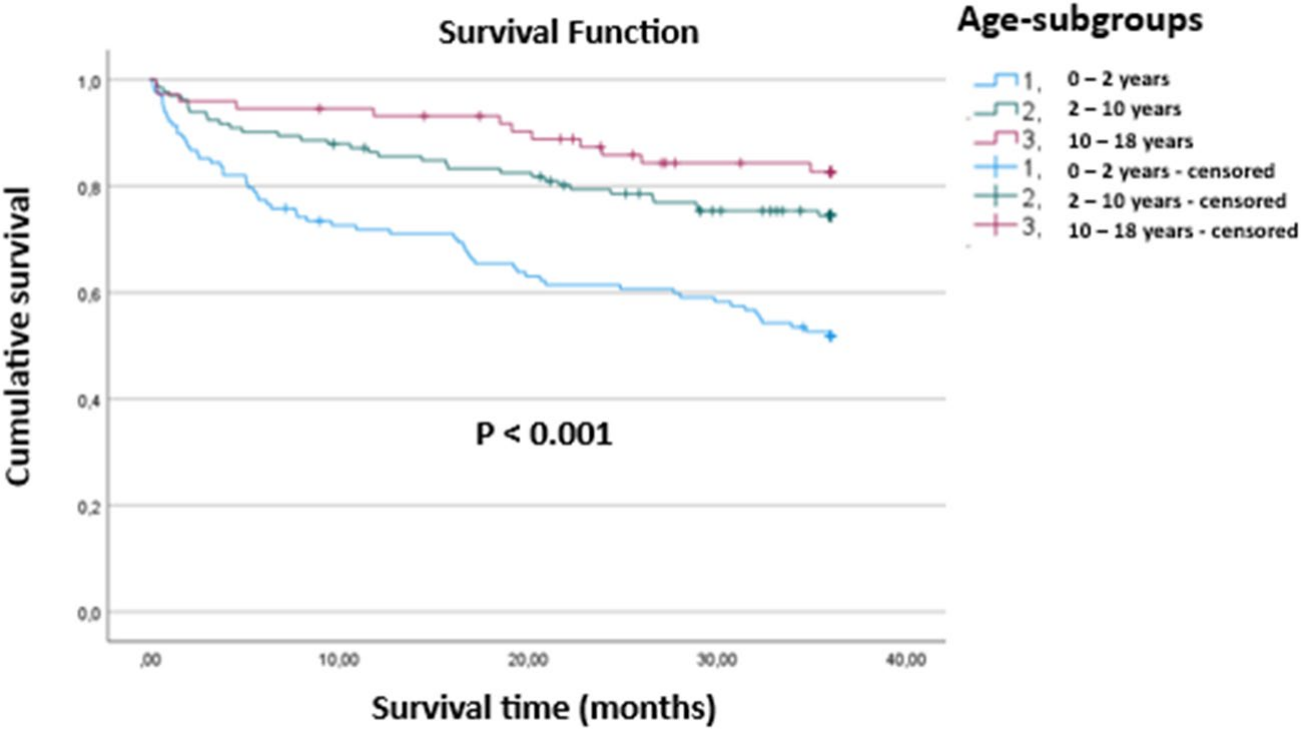
Kaplan–Meier Survival Analysis of proGAV and proGAV 2.0 Valve Survival Across Age Groups

Our study shows a clear distinction in valve survival between these age groups, with statistical analysis confirming significant differences in survival distributions (***p* < 0.001**). The Kaplan–Meier survival curves (Fig. 2) compare the survival rates of the proGAV and proGAV 2.0 valves across different age groups (under 2 years, 2–10 years, and 10–18 years).

## Discussion

In our study, we compared the clinical performance of proGAV and proGAV 2.0 valves in a pediatric population with hydrocephalus, focusing on postoperative complications, valve adjustments, and survival rates. Our findings revealed that the proGAV 2.0 valve exhibited superior performance compared to the proGAV valve, particularly with respect to early postoperative complications and valve adjustment difficulties. These results are consistent with existing literature, but there are notable similarities and differences when compared to other studies examining the use of these valves.

Brunner et al. conducted a retrospective study comparing the revision-free survival rates of proGAV and proGAV 2.0 valves, reporting that both valve systems showed similar revision-free survival rates over a 30-month follow-up period[2]. Our results align with this, as we found no statistically significant difference in overall survival rates between the proGAV and proGAV 2.0 valves (*p* = 0.289). However, Brunner et al. found that the implant duration for proGAV was significantly longer than for proGAV 2.0 (***p* = 0.004**)[2], which contrasts with our findings where proGAV 2.0 demonstrated a longer time to explant (8.2 ± 11.9 months) compared to proGAV (4.5 ± 8.8 months, ***p* = 0.001**). This discrepancy may be attributed to differences in patient populations or follow-up periods. Additionally, they focused on a subgroup of valve-only revisions, which could explain the longer implant duration for proGAV in their cohort[2].

In terms of revision rates, Busse et al. also found no significant differences between the two valves regarding overall revision rates, 1-year revision rates, and revision-free survival[3]. However, they observed that pediatric patients experienced a higher total revision rate with proGAV compared to proGAV 2.0 (***p* = 0.0110**), which is consistent with our findings of fewer valve adjustment difficulties (18.1% vs. 31.2%, ***p* = 0.011**) and spontaneous adjustments (23.8% vs. 33.0%, ***p* = 0.041**) in the proGAV 2.0 group. Furthermore, they reported that revision surgeries were more frequent in pediatric patients, particularly with proGAV valves[3], which is similar to our observation of a higher early complication rate in the proGAV group (22.0% vs. 9.4%, ***p* = 0.002**). These findings support the notion that proGAV 2.0 offers improved stability and ease of management, especially in younger patients who are prone to complications.

Hall et al. conducted a single-center analysis focusing on the efficacy and safety of the proGAV 2.0 valve, particularly in comparison to fixed-pressure valves[7]. Their study showed that the proGAV 2.0 valve resulted in a significantly lower number of revisions per patient per year (***p* = 0.01**) and a greater mean time to revision compared to fixed-pressure valves (37.1 months vs. 31.0 months, ***p* < 0.01**). While they did not directly compare proGAV to proGAV 2.0, their findings on the benefits of proGAV 2.0 in reducing revisions align with our results, where the proGAV 2.0 valve demonstrated improved survival time and fewer valve-related complications compared to proGAV[7]. Additionally, they reported significant clinical improvements post-implantation[7], which is consistent with our observation of a significantly higher rate of clinical improvement within the first six months in the proGAV 2.0 group (86.3% vs. 71.7%, ***p* = 0.001**). Radiological improvement was also greater in the proGAV 2.0 group (53.5% vs. 41.0%, ***p* = 0.038**), further supporting the effectiveness of this valve in managing hydrocephalus.

One important area where our findings diverge from their study is the mean time to valve revision. While they reported a mean time to revision of 37.1 months with proGAV 2.0[7], our study showed a shorter time to explant (8.2 ± 11.9 months), likely due to differences in patient follow-up duration and inclusion criteria. It is also possible that the smaller sample size and shorter follow-up period in our study could explain the observed discrepancies.

All previous studies have consistently shown a significantly lower survival rate in younger patients (< 2 years of age)[6, 12, 13], and our study reinforces this finding. It highlights that valve survival varies significantly across age groups, with younger patients generally experiencing lower survival rates compared to older children. This underscores the critical role age plays in valve longevity and the increased likelihood of valve revision or explantation in younger patients over time.

### Clinical implications and future directions

Overall, our findings, in conjunction with these studies, suggest that the proGAV 2.0 valve offers significant advantages over the original proGAV valve, particularly in terms of reducing early complications, improving valve stability, and enhancing clinical outcomes. However, certain subgroups of patients, especially those undergoing valve-only revisions, may still be at risk for shorter implant durations with proGAV 2.0.

### Limitations

While our study demonstrates the clinical advantages of the proGAV 2.0 valve, the retrospective nature of the analysis and the use of multiple surgeons with differing levels of experience in valve adjustments may introduce some bias. In particular, variations in valve adjustment skills in the outpatient clinic could have contributed to the observed differences in valve adjustment difficulties between the two groups. Additionally, the small sample size of explantations and adjustments, particularly in the valve-only revision subgroup, limits the generalizability of the findings regarding implant duration.

Further prospective studies with longer follow-up periods and larger sample sizes are needed to validate these findings and explore potential patient-specific factors that may influence valve performance.

## Conclusion

The findings of this study underscore the superior performance of the proGAV 2.0 valve, particularly in terms of complication and maladjustment rates. Patients with the proGAV 2.0 valve showed significantly greater clinical and radiological improvement within the first six months following implantation. Both valve types exhibited comparable survival rates over three years, with 65.3% for the proGAV and 71.3% for the proGAV 2.0. However, the proGAV valve was associated with higher early post-implantation complication rates and significantly more frequent issues with valve adjustments and spontaneous readjustments.

## Data Availability

No datasets were generated or analysed during the current study.
